# Supplementary material for: MoSe2/WS2 heterojunction photodiode integrated with a silicon nitride waveguide for near infrared light detection with high responsivity
Source: Light Sci Appl. 2023 Mar 4;12:60. doi: 10.1038/s41377-023-01088-4 (PMC9984525; doi:10.1038/s41377-023-01088-4)
Supplement: Supplementary file 1 — Supplementary Information for: MoSe2/WS2 heterojunction photodiode integrated with a silicon nitride waveguide for near infrared light detection with high responsivity [file 41377_2023_1088_MOESM1_ESM.docx]

**Supplementary Information for:**

**MoSe_2_/WS_2_ heterojunction photodiode integrated with a silicon nitride waveguide for near infrared light detection with high responsivity**

**Rivka Gherabli^1^, S.R.K.C. Indukuri^1^, Roy Zektzer^1^, Christian Frydendahl^1^, and Uriel Levy^1, *^**

*^1^ Department of Applied Physics, The Faculty of Science, The Center for Nanoscience and Nanotechnology, The Hebrew University of Jerusalem, Jerusalem 91904, Israel.*

*^*^ Corresponding author:* [*ulevy@mail.huji.ac.il*](mailto:ulevy@mail.huji.ac.il)

This Supplementary Information comprises the following Sections:

1. Device characterization.

Figure S1: AFM characterization of the 2D device.

Figure S2: Raman characterization of the 2D device.

1. Temperature measurement. Figure S3.
2. Improved dark current through band gap engineering. Figure S4.
3. Reproducibility of the results for two more devices. Figure S5.
4. Photocurrent versus time. Figure S6.
5. Energy bandgap measurement of the MoSe_2_/WS_2_ heterostructure. Figure S7.
6. Loss propagation in silicon nitride waveguide. Figure S8.
7. Frequency response measurement. Figure S9.

**
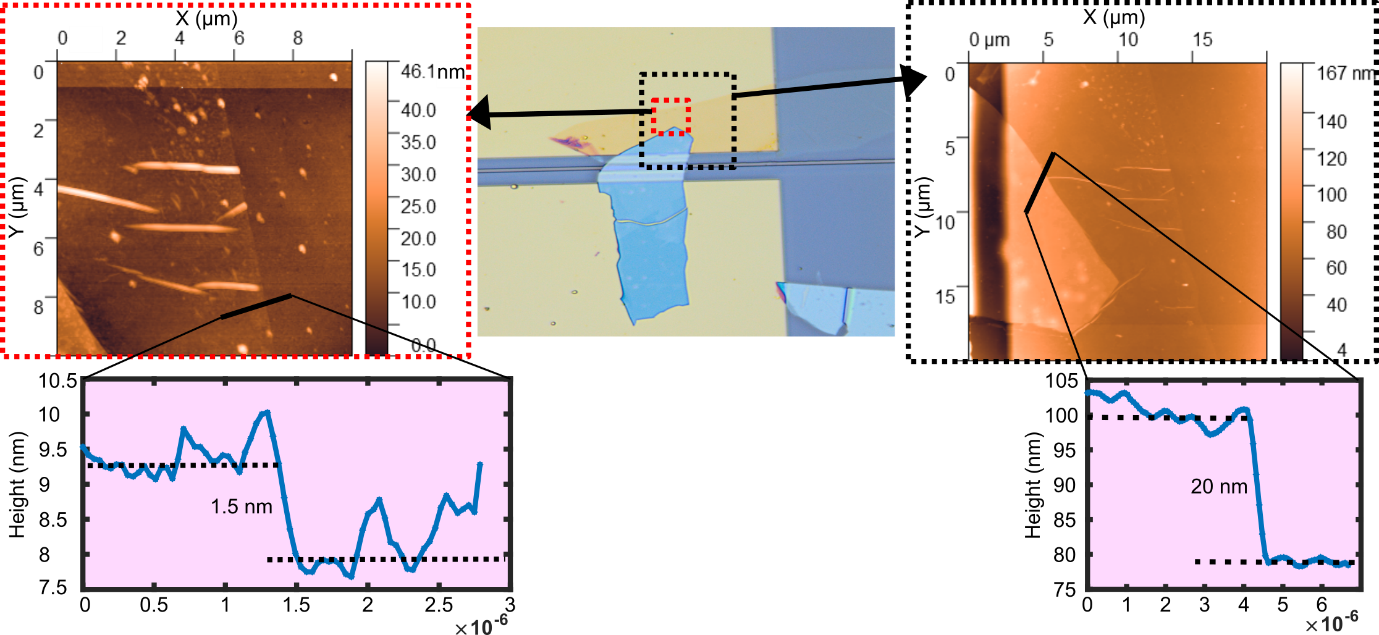
**

**Figure S1. AFM profile.** Figure shows the AFM profile of WS_2_/MoSe_2_ heterojunction in two different areas indicated by the dotted box in the optical image. All the measurements were performed in noncontact mode. The approximated thickness of the MoSe_2_ and WS_2_ is around 20 nm and 1.5 nm respectively.


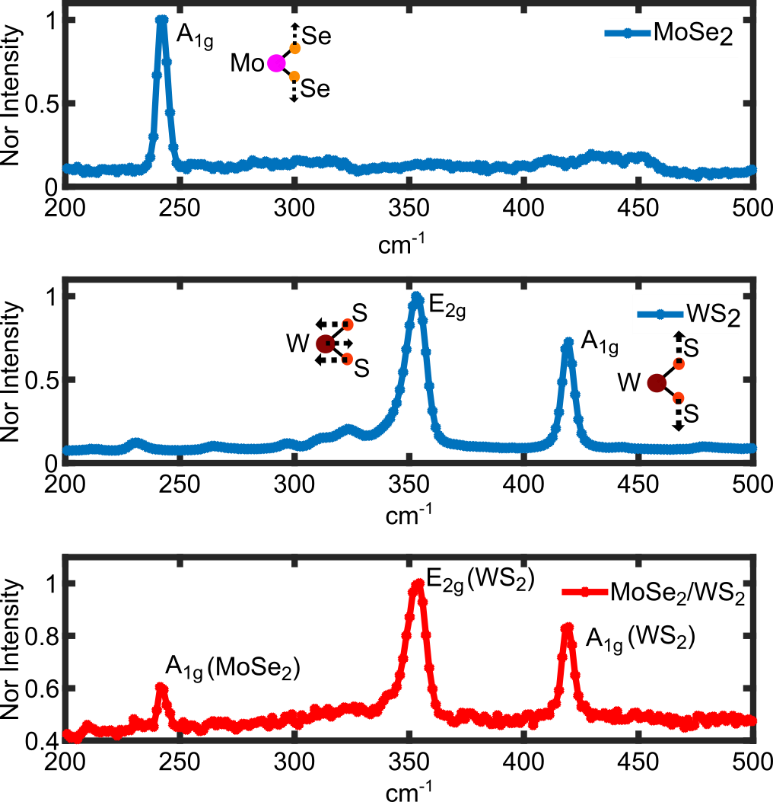


**Figure S2. Raman scattering spectroscopy**. The Raman scattering intensity plots for the MoSe_2_/WS_2_ device. Different peaks are assigned consistently with the previously reported values. In the case of the WS2 layer, *A′/A_1g_* is an out-of-plane phonon mode in which the top and bottom ‘S’ atoms vibrate in opposing directions, while *E′/ E_2g_* is an in-plane mode where the ‘W’ atoms vibrate out-of-phase with the chalcogen atoms. Similarly, in the case of MoSe2 bulk, *A′/A_1g_* is the only active mode where the top and bottom ‘Se’ atoms vibrate in opposing directions. In the case of the heterostructure region, we see Raman scattering from both layers.


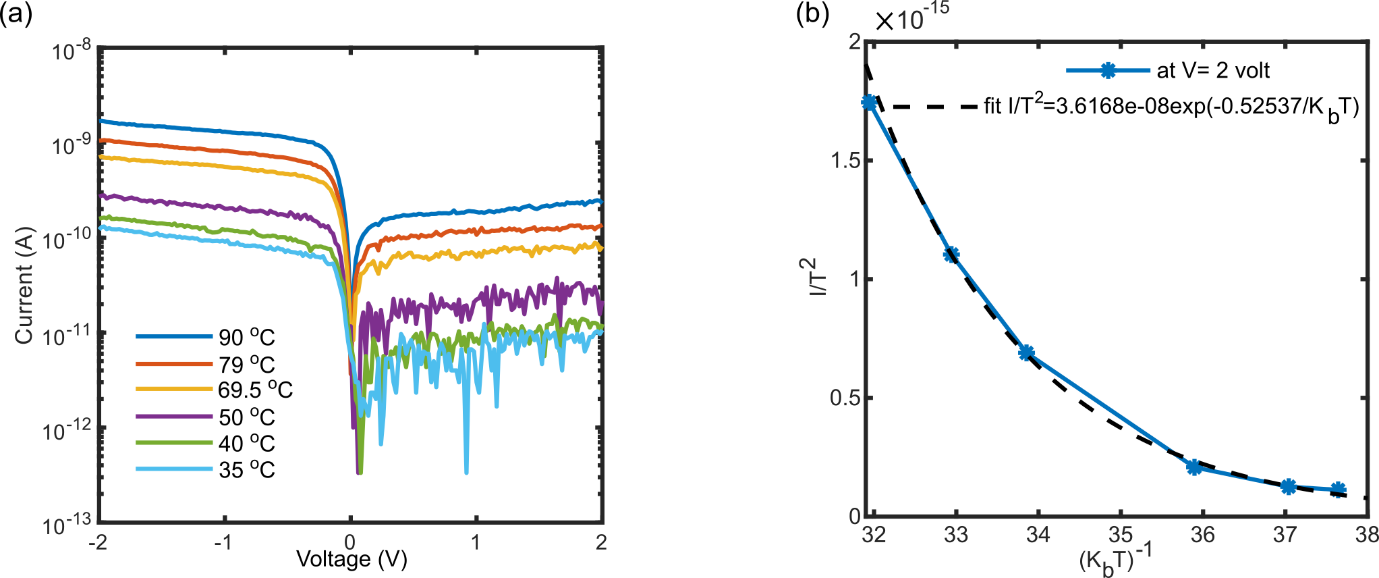


**Figure S3. Temperature measurement.** (a) *I-V* characteristics of the MoSe_2_/WS_2_ heterojunction under different temperature. (b) Normalized inverse temperature square versus the inverse of the thermal energy. The thermionic emission is defined as$J=A٭T^{2}exp \left( {-Ew}/{K_{b}T} \right)$, where *E_W_* = 0.53 eV is the effective barrier height, extracted as a fitting parameter of the bandgap discontinuity at the MoSe_2_/WS_2_ junction interface.

**
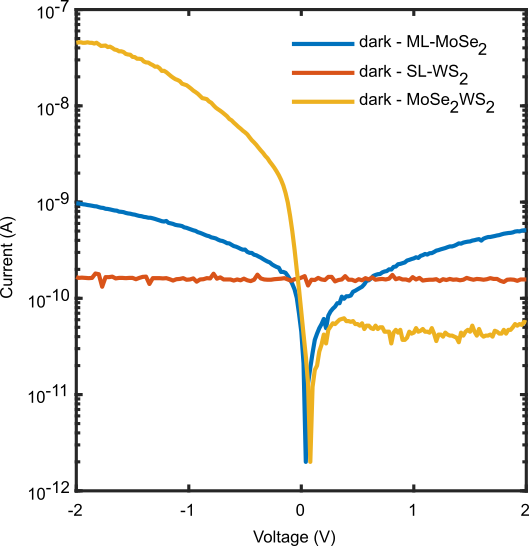
**

**Figure S4. Effect of heterojunction on dark current.** Dark *I-V* curves of multilayer MoSe2, single layer WS2, and the MoSe2/WS2 heterostructure. Only MoSe2 photodetector shows no rectification and large dark current (~ 0.5 nA) whereas with the heterojunction we see the rectification behavior with reduced dark current (~ 40 pA).


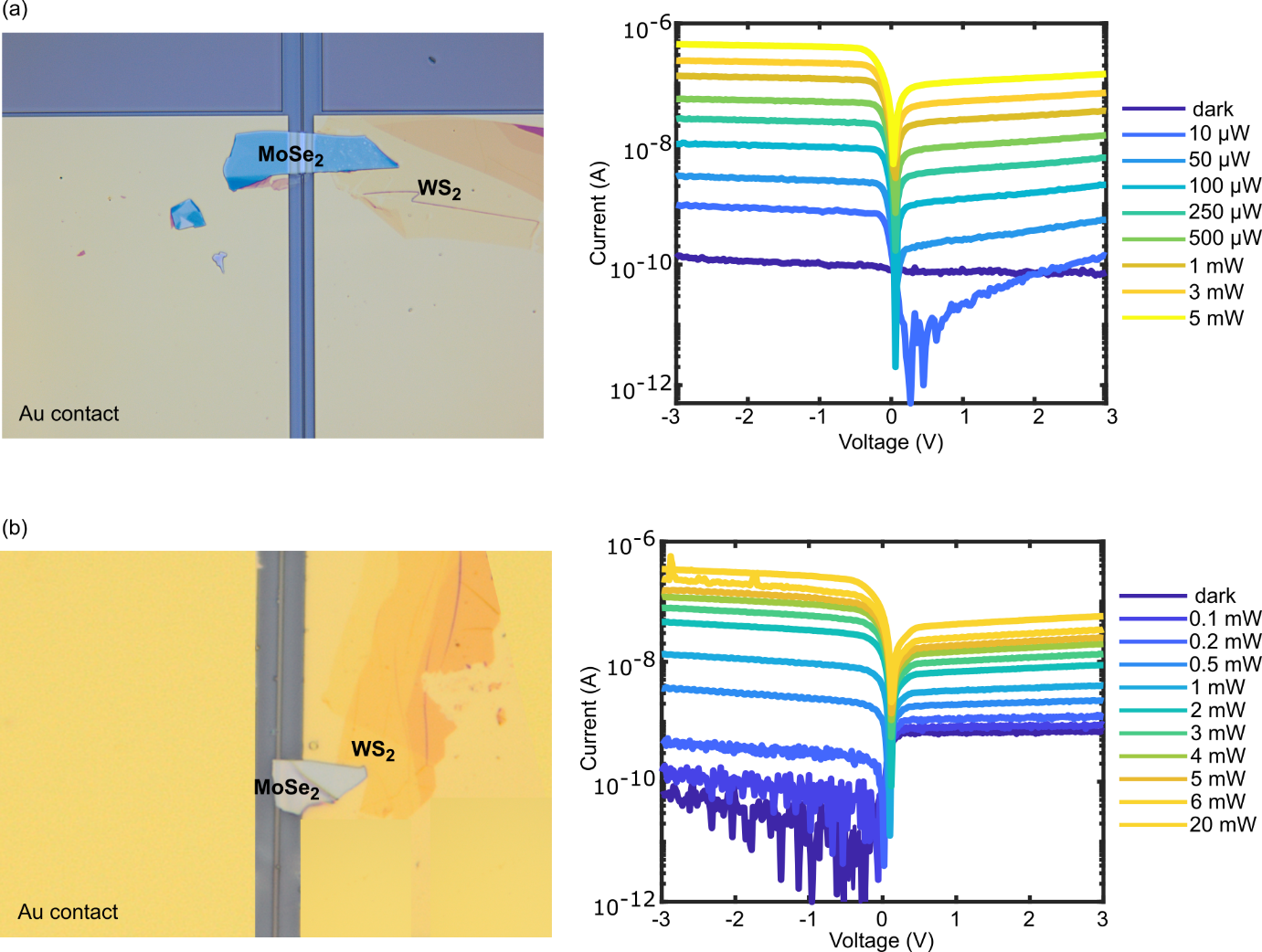


**Figure S5. Other devices characterization.** (a), (b) Optical microscope image and *I-V* characteristics of two other MoSe_2_/WS_2_ heterojunction. **Please note that the optical microscope image in (b) has been took after the measurement, while the MoSe2 multi-layers broke after measurement.**


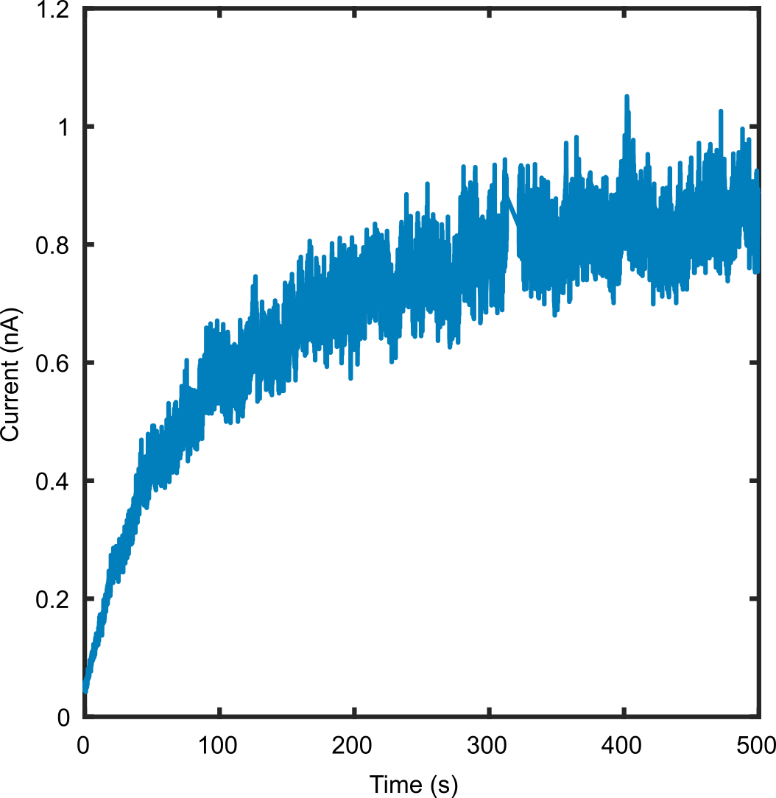


**Figure S6. Photocurrent versus time.**

**
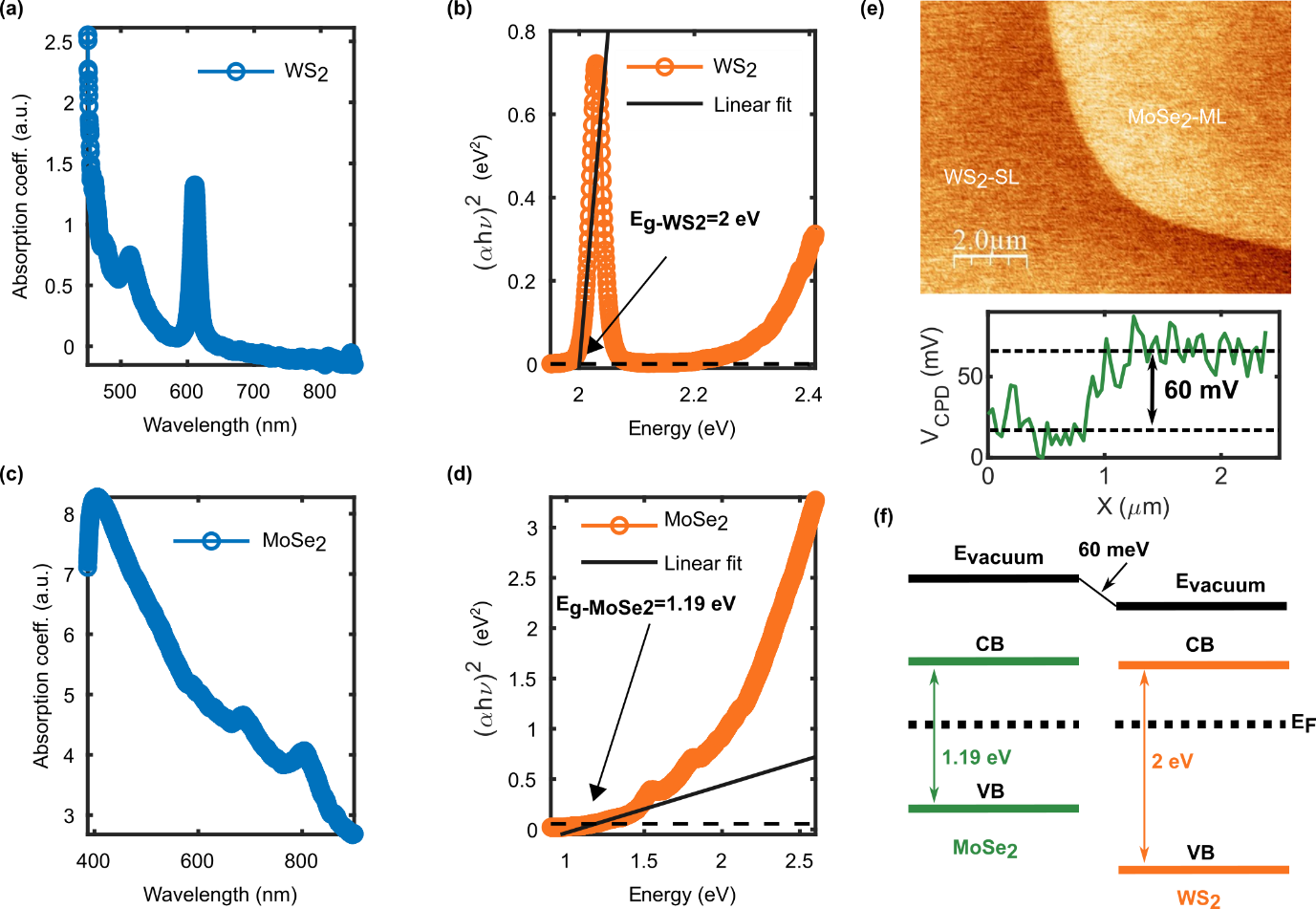
**

**Figure S7. Energy bandgap measurement of the MoSe2/WS2 heterostructure. (a)-(b)** Absorption coefficient and Tauc plot of the single layer of WS_2_. The bandgap energy was experimentally determined to be 2 eV. **(c)-(d)** Absorption coefficient and Tauc plot of bulk MoSe2. The bandgap energy was experimentally determined to be 1.19 eV. **(e)** KPFM measurement. Contact potential difference image and line profile of the MoSe2/WS2 heterojunction. **(f)** schematic of the bulk MoSe_2_, and single layer WS_2_ energy bandgap. The difference in the vacuum energy level between the two materials is about 60 meV.


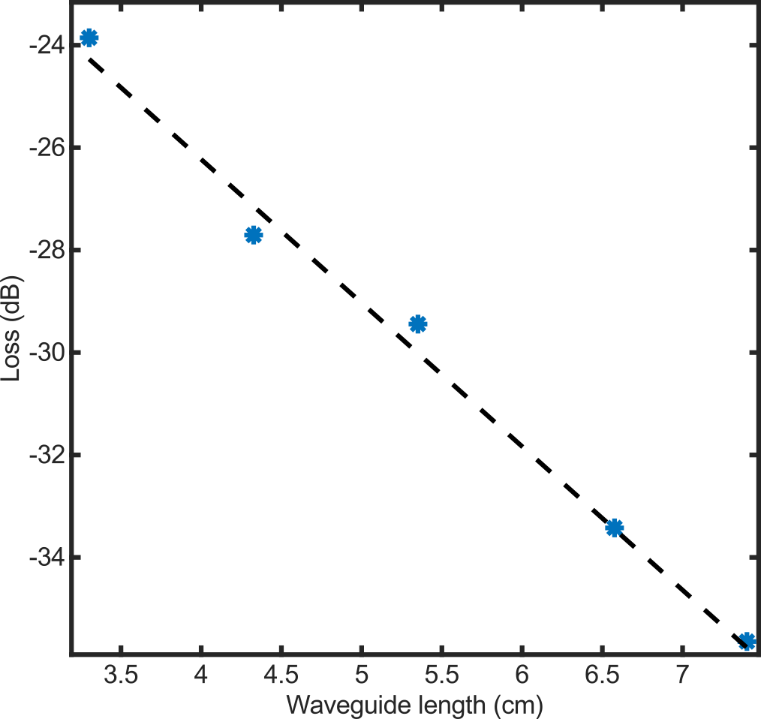


**Figure S8. Loss propagation in silicon nitride waveguide.**


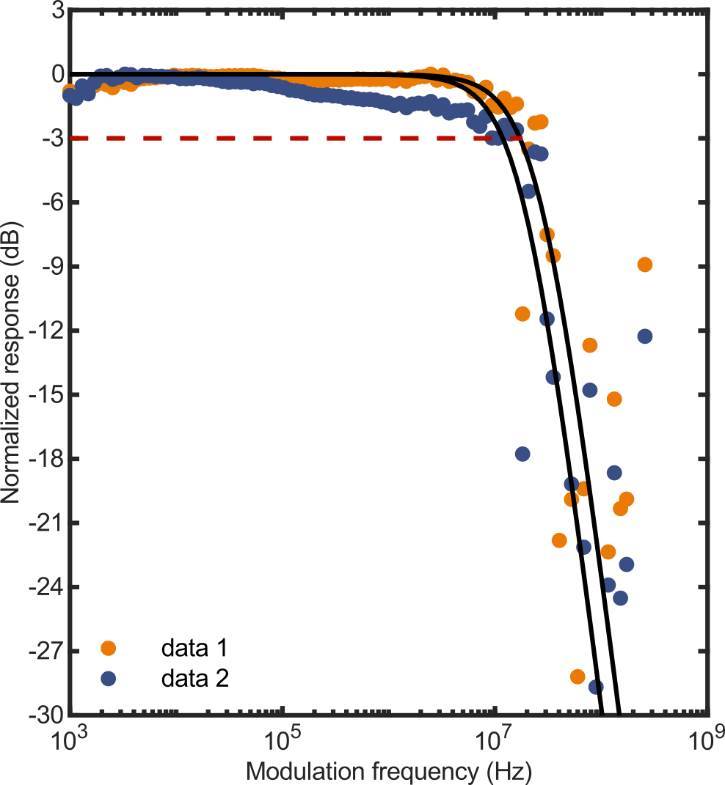


**Figure S9. Frequency response measurement.** Two consecutive measurements are presented. Results show 3 dB cut-off around 20 MHz.
